# Novel ^89^Zr cell labeling approach for PET-based cell trafficking studies

**DOI:** 10.1186/s13550-015-0098-y

**Published:** 2015-03-28

**Authors:** Aditya Bansal, Mukesh K Pandey, Yunus E Demirhan, Jonathan J Nesbitt, Ruben J Crespo-Diaz, Andre Terzic, Atta Behfar, Timothy R DeGrado

**Affiliations:** Department of Radiology, Mayo Clinic, Rochester, 55905 MN USA; Division of Cardiovascular Diseases, Mayo Clinic, Rochester, 55905 MN USA

**Keywords:** Zirconium-89, PET, Cell labeling, *In vivo* cell tracking

## Abstract

**Background:**

With the recent growth of interest in cell-based therapies and radiolabeled cell products, there is a need to develop more robust cell labeling and imaging methods for *in vivo* tracking of living cells. This study describes evaluation of a novel cell labeling approach with the positron emission tomography (PET) isotope ^89^Zr (*T*_1/2_ = 78.4 h). ^89^Zr may allow PET imaging measurements for several weeks and take advantage of the high sensitivity of PET imaging.

**Methods:**

A novel cell labeling agent, ^89^Zr-desferrioxamine-NCS (^89^Zr-DBN), was synthesized. Mouse-derived melanoma cells (mMCs), dendritic cells (mDCs), and human mesenchymal stem cells (hMSCs) were covalently labeled with ^89^Zr-DBN via the reaction between the NCS group on ^89^Zr-DBN and primary amine groups present on cell surface membrane protein. The stability of the label on the cell was tested by cell efflux studies for 7 days. The effect of labeling on cellular viability was tested by proliferation, trypan blue, and cytotoxicity/apoptosis assays. The stability of label was also studied in *in vivo* mouse models by serial PET scans and *ex vivo* biodistribution following intravenous and intramyocardial injection of ^89^Zr-labeled hMSCs. For comparison, imaging experiments were performed after intravenous injections of ^89^Zr hydrogen phosphate (^89^Zr(HPO_4_)_2_).

**Results:**

The labeling agent, ^89^Zr-DBN, was prepared in 55% ± 5% decay-corrected radiochemical yield measured by silica gel iTLC. The cell labeling efficiency was 30% to 50% after 30 min labeling depending on cell type. Radioactivity concentrations of labeled cells of up to 0.5 MBq/10^6^ cells were achieved without a negative effect on cellular viability. Cell efflux studies showed high stability of the radiolabel out to 7 days. Myocardially delivered ^89^Zr-labeled hMSCs showed retention in the myocardium, as well as redistribution to the lung, liver, and bone. Intravenously administered ^89^Zr-labeled hMSCs also distributed primarily to the lung, liver, and bone, whereas intravenous ^89^Zr(HPO_4_)_2_ distributed to the liver and bone with no activity in the lung. Thus, the *in vivo* stability of the radiolabel on the hMSCs was evidenced.

**Conclusions:**

We have developed a robust, general, and biostable ^89^Zr-DBN-based cell labeling strategy with promise for wide applications of PET-based non-invasive *in vivo* cell trafficking.

## Background

With the growth of interest in cell-based therapies, there is a need to develop more sensitive, robust, and quantitative imaging methods for *in vivo* tracking of living cells. A number of radioisotopic cell labeling methods have traditionally been used for single-photon emission computerized tomography (SPECT) and positron emission tomography (PET) imaging-based cell tracking [1]. However, a PET-based approach would offer superior quantification and imaging sensitivity characteristics over a SPECT-based approach, which are critical for tracking of small numbers of administered cells [1]. In this regard, ^89^Zr has emerged as an attractive PET radionuclide for cell labeling applications due to its high spatial resolution and 78.4-h half-life that may allow monitoring of administered cells up to a 2- to 3-week period.

A variety of cell labeling strategies have been forwarded, including transport of a radiometal (^111^In, ^99m^Tc, ^64^Cu, ^89^Zr) into cells in conjunction with oxine, hexamethylpropyleneamine oxime (HMPAO), pyruvaldehyde-bis(N4-methylthiosemicarbazone) (PTSM), or protamine sulfate, or antibody-based labeling (Table 1) [1-11]. In the transport approach, after entry into the cell, the radiometal dissociates and binds to a variety of intracellular biomolecules. The major drawback of this approach is that appreciable efflux of sequestered radioactivity is observed post-labeling. The extent of efflux has been as high as 70% to 80% in 24 to 96 h as reported for ^111^In-oxine-labeled lymphocytes [4], ^111^In-oxine-labeled hematopoietic progenitor cells [5], and ^64^Cu-PTSM-labeled C6 glioma cells [7]. Recently, ^89^Zr-oxine has been reported as a labeling molecule but like ^111^In-oxine, it also undergoes efflux (10% to 29% at 24 h in macrophages, breast cancer cells, and myeloma cells [9] and 70% to 80% at 24 h in natural killer cells [10]). Efflux of radiolabel significantly limits monitoring cell trafficking over longer observational periods. Cells have also been labeled with ^18^ F-FDG [12-16] (*T*_1/2_ = 109.8 min), ^99m^Tc-HMPAO [17] (*T*_1/2_ = 6 h), and ^64^Cu-labeled anti-CD45 [8] (*T*_1/2_ = 12.7 h), but the short half-lives of these radioisotopes limit their utility for cell tracking to shorter observational periods. An alternative antibody-based stem cell labeling method employed ^89^Zr-labeled anti-CD45 for *ex vivo* labeling of stem cells expressing CD45 membrane protein. However, this radiotracer yielded poor *in vivo* imaging characteristics, possibly due to insufficient CD45 molecules on the plasma membrane of stem cells [8].

**Table 1:** **Present direct radioisotopic cell labeling methods**

**Isotope-compound/** ***T***_**1/2**_	**Cells labeled**	**Labeling and imaging characteristics**	**Reference**
^111^In-oxine/67.4 h	Leukocytes	Approximately 80% cell labeling yield in 30 min	[2-6]
	Lymphocytes	Significant efflux rate reported in lymphocytes (approximately 70% effluxed in 24 h) and HPCs (approximately 75% effluxed in 96 h)	
	HPCs	Suboptimal image quality and sensitivity	
^64^Cu-PTSM/12.7 h	C6 glioma cells	70% to 85% cell labeling yield in 5 h	[7]
		Significant efflux rate from cells (approximately 80% effluxed in 24 h)	
^64^Cu-TETA- or ^89^Zr-DFO-antiCD45/12.7 h (^64^Cu), 78.4 h (^89^Zr)	hPBSCs	Binds to only CD45 membrane protein expressing cells	[8]
		Approach was suboptimal possibly due to insufficient CD45 molecules on the plasma membrane of stem cells	
^89^Zr-oxine/78.4 h	Myeloma cells and natural killer cells	Approximately 32% cell labeling yield in 30 min	[9,10]
		Significant efflux rate reported for myeloma cells (29% effluxed in 24 h) and natural killer cells (70% to 80% effluxed in 7 days)	
		Loss of cell viability possibly due to oxine exposure	
^89^Zr-protamine sulfate/78.4 h	Dendritic cells and T lymphocytes	Approximately 34% cell labeling yield (dendritic cells) in 30 min	[11]
		Approximately 12% cell labeling yield (T lymphocytes) in 30 min	
		Weakly binds to non-specific intracellular biomolecules	
		Efflux rate not reported	

HPCs, hematopoietic progenitor cells; hPBSCs, human peripheral blood stem cells.

**Figure 1:**
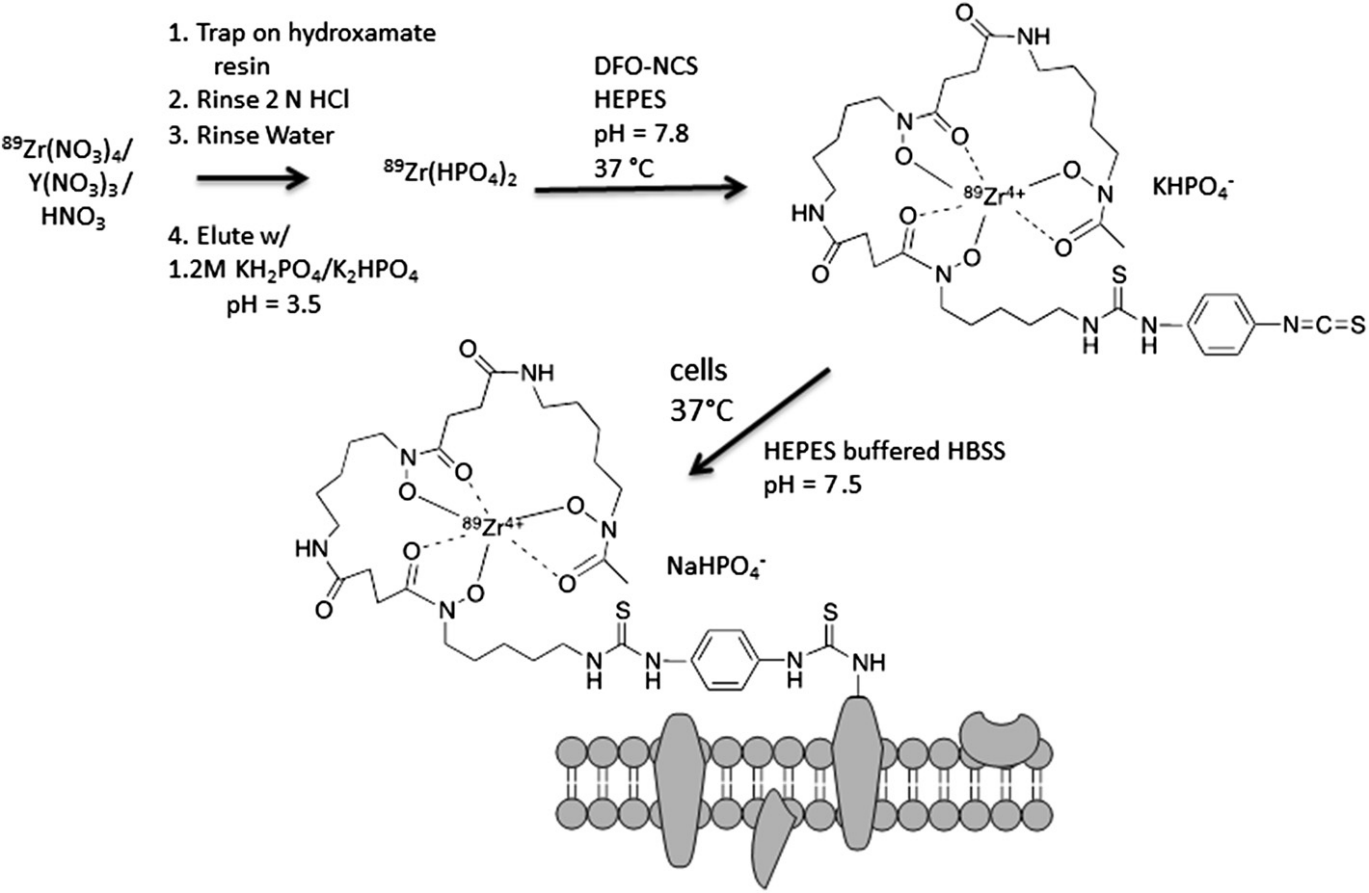
**Scheme for synthesis of** ^**89**^ **Zr-DBN and cell labeling.**

In this study, we propose a novel cell labeling strategy that covalently binds a ^89^Zr-DFO-labeled agent to cell surface proteins independent of cell type. The novel method employs the two-step process (Figure 1): 1) preparation of ^89^Zr-labeled *p*-isothiocyanato-benzyl-desferrioxamine (^89^Zr-DBN) and 2) random labeling of primary amines of cell surface proteins with ^89^Zr-DBN. We have evaluated this labeling strategy in three cell types: mouse melanoma cells (mMCs), human mesenchymal stem cells (hMSCs), and mouse dendritic cells (mDCs). The labeled cells were evaluated for 7 days post-labeling for label retention and probable changes in cell proliferation, cell viability, and degree of apoptosis in radiolabeled cells as compared to their unlabeled counterparts. Out of these, labeled hMSCs were further tested for imaging characteristics and stability of radiolabel in an *in vivo* mouse model.

## Methods

### Cell culture

B16-F10 mMCs from ATCC, Manassas, VA, USA, hMSCs from patients, and JAWSII mDCs from ATCC, Manassas, VA, USA, were used for evaluating the ^89^Zr-DBN-based labeling method. The mMCs and hMSCs were cultured in complete Dulbecco’s modified Eagle’s medium (DMEM) (DMEM + 10% FBS), and mDCs were cultured in complete alpha MEM (alpha MEM + 4 mM L-glutamine + 1 mM sodium pyruvate + 5 ng/mL murine GM-CSF + 20% FBS). The cultures were maintained in a humidified cell culture chamber (21% O_2_, 74% N_2_, 5% CO_2_) at 37°C.

### Production and isolation of ^89^Zr

^89^Zr^4+^ was produced in aqueous solution through the ^89^Y(*p,n*)^89^Zr nuclear reaction using a solution target containing yttrium nitrate and dilute nitric acid [18]. The ^89^Zr^4+^ was isolated from ^89^Y^3+^ using a hydroxamate resin-based purification method [18,19] with the exception that the final elution of ^89^Zr^4+^ off the hydroxamate resin was performed with an appropriate volume of 1.2 M K_2_HPO_4_/KH_2_PO_4_ buffer (pH 3.5). The K_2_HPO_4_/KH_2_PO_4_ buffer was allowed to sit on the column for 30 min before elution to promote release of ^89^Zr as zirconium hydrogen phosphate, ^89^Zr(HPO_4_)_2_, from the column. The elution percentage of ^89^Zr from the column was approximately 89% collected in four fractions of 0.5 mL each.

### Synthesis of ^89^Zr-DBN

The eluted ^89^Zr(HPO_4_)_2_ solution (120 μL) was neutralized to pH 7.8 with 100 μL 1 M HEPES-KOH buffer (pH 7.5) and 65 μL 1 M K_2_CO_3_. To this, 4 μL 5 mM DFO-Bz-NCS in DMSO (Macrocylics, Dallas, TX, USA) was added, and chelation of ^89^Zr^4+^ proceeded at 37°C for 1 h in a thermomixer at 550 rpm. Chelation efficiency was determined by silica gel iTLC (Agilent Technologies, Santa Clara, CA, USA) with 50 mM DTPA pH 7 as the mobile phase. ^89^Zr-DBN showed an *R*_*f*_ = 0, whereas ^89^Zr(HPO_4_)_2_ had an *R*_*f*_ = 0.9.

### Labeling of cells with ^89^Zr(HPO_4_)_2_ and ^89^Zr-DBN

The adherent cells were trypsinized and washed once with PBS and twice with HEPES buffered GIBCO Hanks Balanced Salt solution buffered (Thermo Fisher, Waltham, MA, USA) (H-Hank’s Balanced Salt Solution (HBSS), pH 7.5. The cell labeling reaction was performed with approximately 6 × 10^6^ cells in 500 uL H-HBSS at pH 7.5. To this, either 100 μL ^89^Zr(HPO_4_)_2_ (approximately 6 MBq) or 100 uL ^89^Zr-DBN (approximately 6 MBq) was added and was allowed to incubate at 37°C for 30 min on a shaker for cell labeling. After incubation, the cells were washed four times with appropriate volume of complete medium. The final labeling efficiency was calculated from the radioactivity bound to cells after all the washes.

### Incorporation of ^89^Zr-DBN in protein fraction

To understand the subcellular localization of the label, incorporation of ^89^Zr-DBN into different protein fractions in mMCs, hMSCs, and mDCs was evaluated using a subcellular protein fractionation kit (Piercenet Thermo Scientific, Waltham, MA, USA) at days 1, 4, and 7 post-labeling*.* The cytosolic proteins, hydrophobic membrane proteins, nuclear proteins, and cytoskeletal proteins were isolated, and each protein fraction was counted for radioactivity using a 2480 Wizard^2^ automatic gamma counter (PerkinElmer, Waltham, MA, USA).

### Efflux of ^89^Zr-DBN from labeled cells

To determine cellular efflux, 0.3 × 10^6^^89^Zr-labeled cells were plated into each well of a six-well culture plate. The medium was replaced with fresh medium daily for 7 days, and radioactivity in the replaced medium was counted. For mDCs with mix of adherent and suspension cells, the plate was centrifuged at 1,000 rpm for 10 min before replacing the medium to avoid loss of unattached ^89^Zr-labeled cells.

### CyQUANT cellular proliferation assay

The effect of radiolabeling on cellular proliferation was assessed by the CyQUANT DNA content assay (Thermo Fisher, Waltham, MA, USA). A known number of unlabeled and ^89^Zr-labeled cells (approximately 10^4^ cells/well) were plated in 21 wells of a 96-well culture plate and maintained at 37°C in a CO_2_ incubator. The amount of DNA in each well was quantified from absorbance values as a surrogate marker of the number of cells present. The culture medium was replaced daily. The CyQUANT assay was performed for three wells per day over 5 days.

### Trypan blue exclusion assay cellular viability test

The effect of labeling on cellular viability was assessed using trypan blue exclusion assay test within 1 h of labeling, third and seventh day post-labeling. The culture medium was replaced daily and maintained at 37°C in a CO_2_ incubator. Unlabeled cells served as control.

### ApoTox-Glo viability/cytotoxicity and apoptosis assay

The effect of radiolabeling on cellular viability was also assessed using the ApoTox-Glo viability, cytotoxicity, and caspase 3/7 apoptosis assay (Promega Corporation, Madison, WI, USA). Unlabeled cells served as control. A known number of unlabeled and ^89^Zr-labeled cells (approximately 10^4^/well) were plated in a 96-well culture plate. The culture medium was replaced daily and maintained at 37°C in a CO_2_ incubator. At day 7, cell viability, cytotoxicity, and apoptosis were quantified in triplicate using the ApoTox-Glo assay. As positive controls, cells were incubated with 30 μg/mL digitonin for 30 min for the viability and cytotoxicity assays, while 2 μM staurosporine was added for 16 h for the caspase 3/7 dependent apoptosis assay.

### PET imaging and ex-vivo biodistribution of ^89^Zr-labeled cells and ^89^Zr(HPO_4_)_2_

Experiments were performed with 2-month-old athymic nude Foxn1^nu^ mice (Harlan Laboratories, Inc., Indianapolis, IN, USA). ^89^Zr(HPO_4_)_2_ (approximately 0.074 MBq) or ^89^Zr-labeled cells (2 × 10^5^ cells with radioactivity concentration approximately 0.37 MBq/1 × 10^6^ cells) were injected intravenously through a tail vein. On days 2, 4, and 7, the mice were anesthetized under 1% to 2% isoflurane and underwent PET imaging using a small animal PET/X-RAY system (Sofie BioSystems Genesys4, Culver City, CA, USA). At day 7, the mice were sacrificed and tissues were extracted and radioactivity counted using a gamma counter to evaluate the biodistribution of ^89^Zr radioactivity. PET images were normalized to units of standardized uptake value (SUV) = (activity concentration in tissue / (injected dose/g whole body wt.)) and presented as a coronal sectional images.

### *In vivo* tracking of stem cell engraftment in ischemia/reperfusion mouse model

Athymic nude Foxn1^nu^ mice (2 months old) were anesthetized under 1% to 2% isoflurane and placed on a heating pad maintained at 37°C. Respiratory and heart rates were monitoring continually. After intubation, mechanical ventilation and intercostal block of bupivacaine and lidocaine, an incision was made in through the fourth or fifth intercostal space for access into the thoracic space, the heart was exposed and the pericardium was incised anterior and parallel to the phrenic nerve. With visualization of the coronary vasculature, the left coronary artery was ligated to induce myocardial ischemia at the anterior wall of the left ventricle. One hour after the coronary ligation, the suture was untied for reperfusion. Myocardial reperfusion was confirmed by color change of the left ventricle and electrocardiographic changes. During reperfusion, ^89^Zr-labeled cells (2 × 10^5^ cells with radioactivity concentration approximately 0.37 MBq/10^6^ cells) were injected at four sites within the ischemic region. After myocardial injection, the intercostal space, the chest musculature, and the skin were closed with a 7-0 Ethilon suture. The animals were imaged at day 2, day 5, and day 7 using small animal PET/X-RAY system (Sofie BioSystems Genesys4, Culver City, CA, USA). At day 7, the mice were sacrificed and tissues were extracted and radioactivity counted using gamma counter to evaluate cell trafficking. PET images were normalized to units of SUV and presented as coronal sectional images.

### Statistical analysis

The data were compared using unpaired Student’s *t*-test analyses. Differences were regarded as statistically significant for *p* < 0.05.

## Results

### Synthesis of ^89^Zr-DBN and cell labeling studies

^89^Zr hydrogen phosphate was readily chelated by DFO-NCS to form ^89^Zr-DBN, with radiolabeling efficiency of 55% ± 5% after 1 h of reaction. This reaction mixture was then used directly for labeling of cells. The cell labeling efficiency using ^89^Zr-DBN was approximately 30% to 50% as determined by cell-bound radioactivity. Radioactivity concentrations of 0.50 ± 0.10, 0.47 ± 0.10, and 0.39 ± 0.20 MBq/10^6^ cells were achieved when 6 × 10^6^ cells were incubated for 30 min with approximately 6 MBq ^89^Zr-DBN with mMCs, hMSCs, and mDCs, respectively. In contrast, no cell labeling was observed using ^89^Zr(HPO_4_)_2_*.*

### Cellular proliferation and viability studies

**Figure 2:**
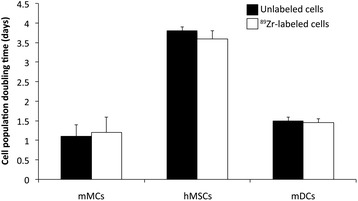
**Comparison of cell population doubling times for** ^**89**^ **Zr-labeled and unlabeled mMCs, hMSCs and mDCs.** The cells were plated at appropriate cell number at day 3, and CyQUANT assay was performed at day 7 post-labeling. No significant differences were observed between radiolabeled and unlabeled cells. Values are shown as mean ± standard deviation, *n* = 3.

The CyQUANT proliferation assay showed no difference in proliferation rate between unlabeled and ^89^Zr-labeled cells (Figure 2). Trypan blue cell viability tests were performed on radiolabeled cells immediately after labeling and up to 7 days post-labeling and compared with unlabeled cells. No change was observed in number of dead cells (blue-stained cells) over live cells (unstained cells) in both ^89^Zr-labeled and unlabeled cells, with percentage of dead cells <5% in all days tested.

### ApoTox-Glo viability/cytotoxicity and apoptosis assay

**Figure 3:**
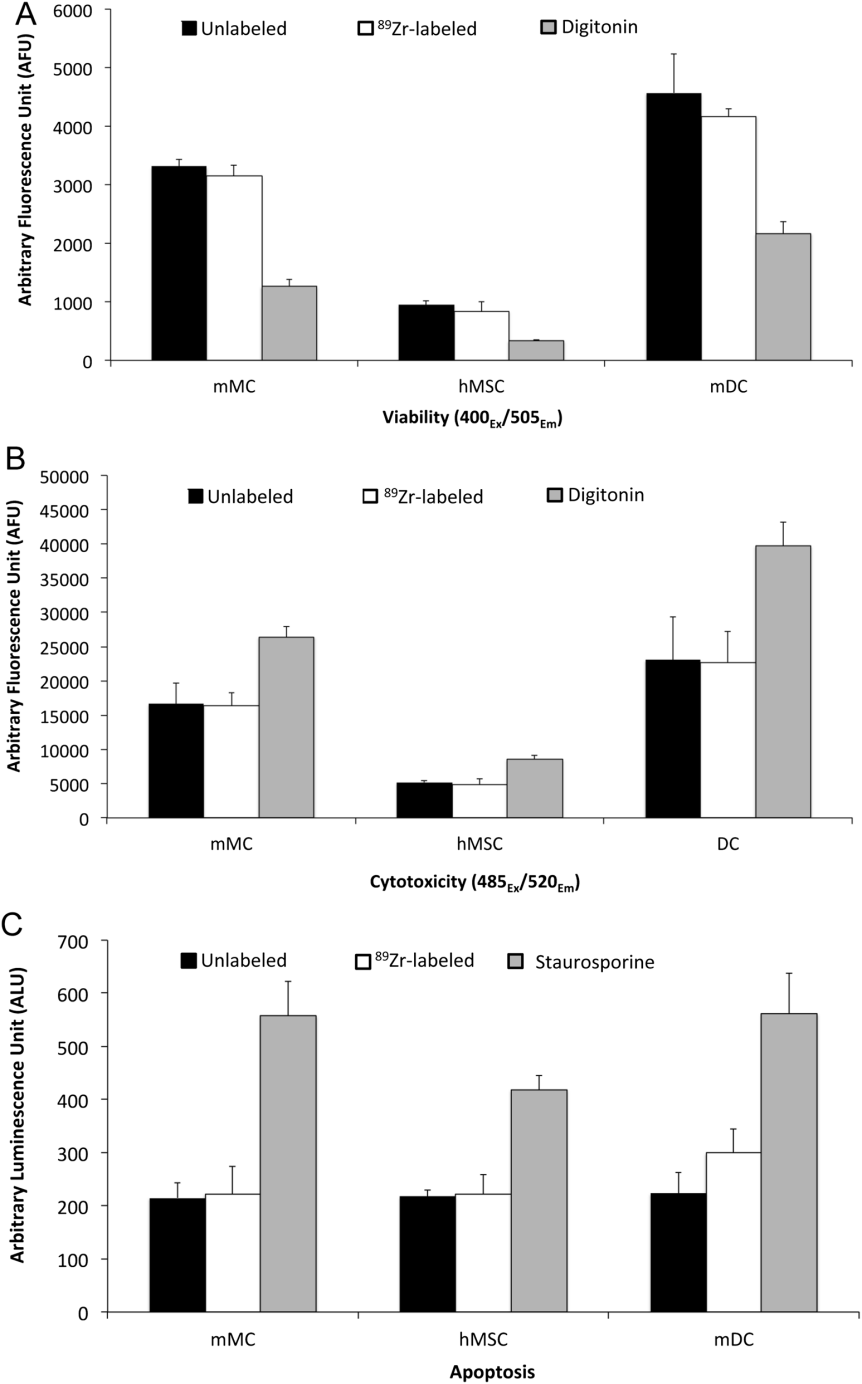
**Assessment of (A) viability, (B) cytotoxicity, and (C) apoptosis in** ^**89**^ **Zr-labeled and unlabeled cells.** No statistically significant differences were observed between ^89^Zr-labeled and unlabeled cells after 7 days of culture with regard to viability, cytotoxicity, or apoptosis. As positive controls, 30 μg/mL digitonin was used for assays **(A)** and **(B)**, and 2 μΜ staurosporine for **(C)**. *p < 0.05 versus assessments in ^89^Zr-labeled and unlabeled cells using unpaired *t*-test. Values are shown as mean ± standard deviation, *n* = 3.

The ApoTox-Glo assay showed no loss in cellular viability and no increase in cytotoxicity or apoptosis in radiolabeled cells as illustrated in Figure 3. Viability was lost, and cytotoxicity enhanced when 30 μg/mL digitonin was added to cells (positive control), and apoptosis was increased with the addition of 2 μM staurosporine (positive control).

### Subcellular distribution of ^89^Zr radioactivity

At days 1, 4, and 7 after ^89^Zr labeling of mMCs, hMSCs, and mDCs, subcellular protein fractionation of the cells was performed. ^89^Zr radioactvity was incorporated predominantly (>99%) in hydrophobic membrane protein fraction of all cell types studied, strongly supporting the proposed mechanism of reaction of ^89^Zr-DBN with cell surface membrane protein to form a stable covalent bond.

### Efflux of ^89^Zr radioactivity from labeled cells

**Figure 4:**
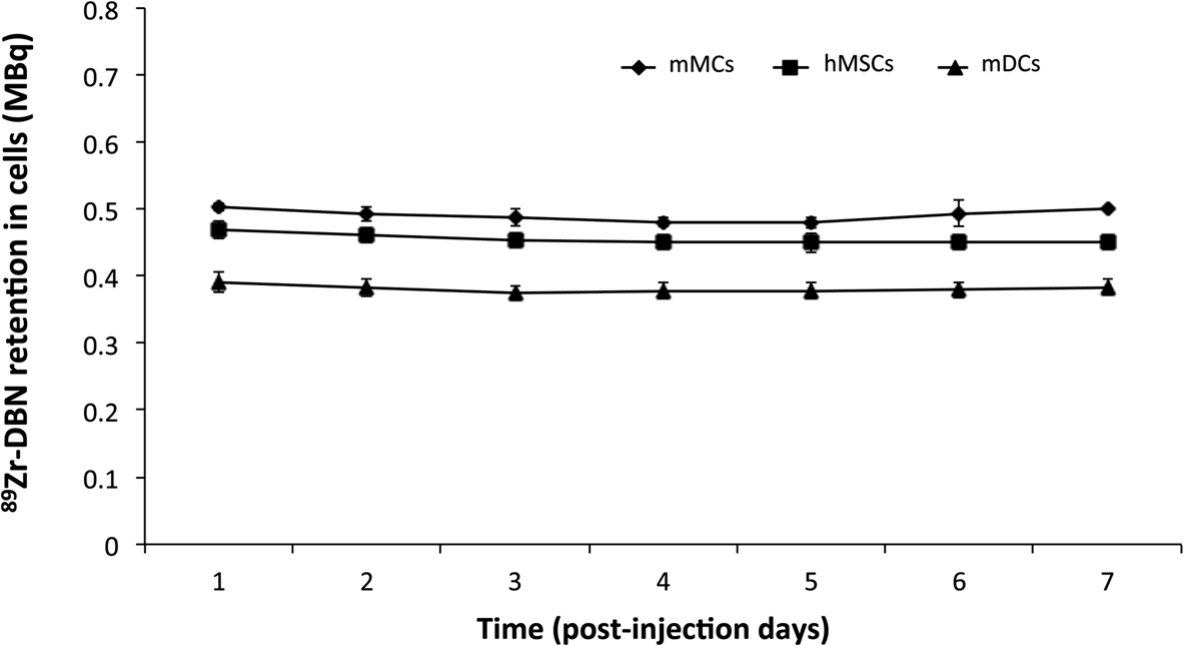
**Retention of** ^**89**^ **Zr in** ^**89**^ **Zr-labeled cells expressed as radioactivity in MBq in the cell population.** The retention value is representing total radioactivity/10^6^ cells in the proliferating cell population. No significant change was observed in retention of ^89^Zr in radiolabeled cells. Values are shown as mean ± standard deviation, *n* = 3.

Retention of ^89^Zr radioactivity by ^89^Zr-DBN-labeled cells was found to be stable in all the cells studied with negligible efflux observed over 7 days post-labeling (Figure 4).

### PET imaging and biodistribution studies in mice with intravenous injections

**Figure 5:**
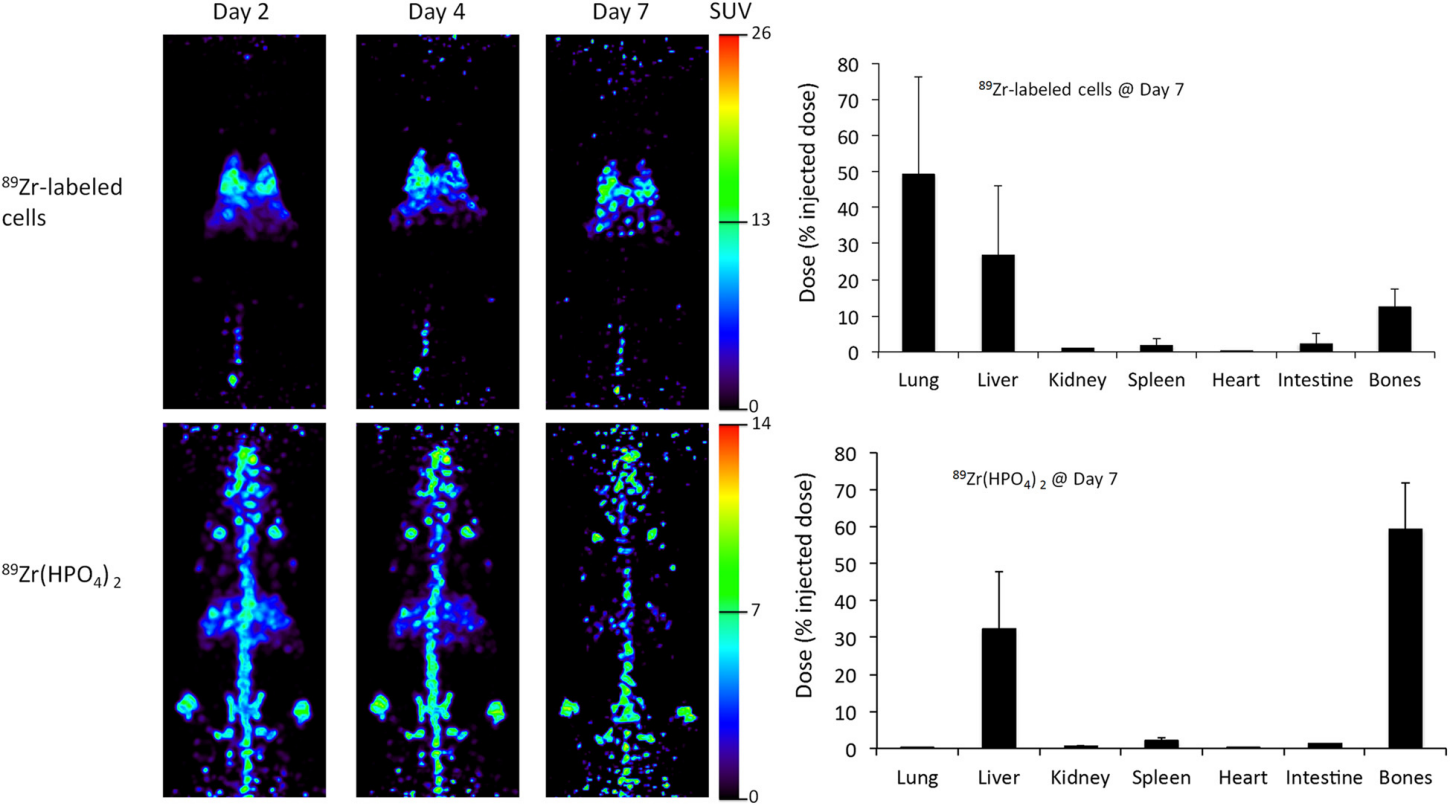
**Representative PET images and biodistribution data of** ^**89**^ **Zr-labeled hMSCs and** ^**89**^ **Zr(HPO**_**4**_ **)**_**2**_ **following intravenous injection.** ^89^Zr-labeled human MSCs (2 × 10^5^ cells with radioactivity concentration approximately 0.37 MBq/10^6^ cells) and ^89^Zr(HPO_4_)_2_ (approximately 0.074 MBq radioactivity) were intravenously injected in athymic mice. Most of the radioactivity was distributed in the lung, liver, and bones following injection of ^89^Zr-labeled hMSCs whereas most of the radioactivity was distributed in the liver and bones following injection of ^89^Zr(HPO_4_)_2_. Values in graphs are shown as mean ± standard deviation, *n* = 3.

PET images and biodistribution data of intravenously administered ^89^Zr-labeled hMSCs and ^89^Zr(HPO_4_)_2_ in healthy mice are shown in Figure 5. The ^89^Zr-labeled hMSCs were concentrated primarily in the lung and liver, followed by the bone. On the other hand, ^89^Zr(HPO_4_)_2_ accumulated in the bone and liver and did not distribute to lung.

### *In vivo* tracking of stem cell engraftment in ischemia/reperfusion mouse model

**Figure 6:**
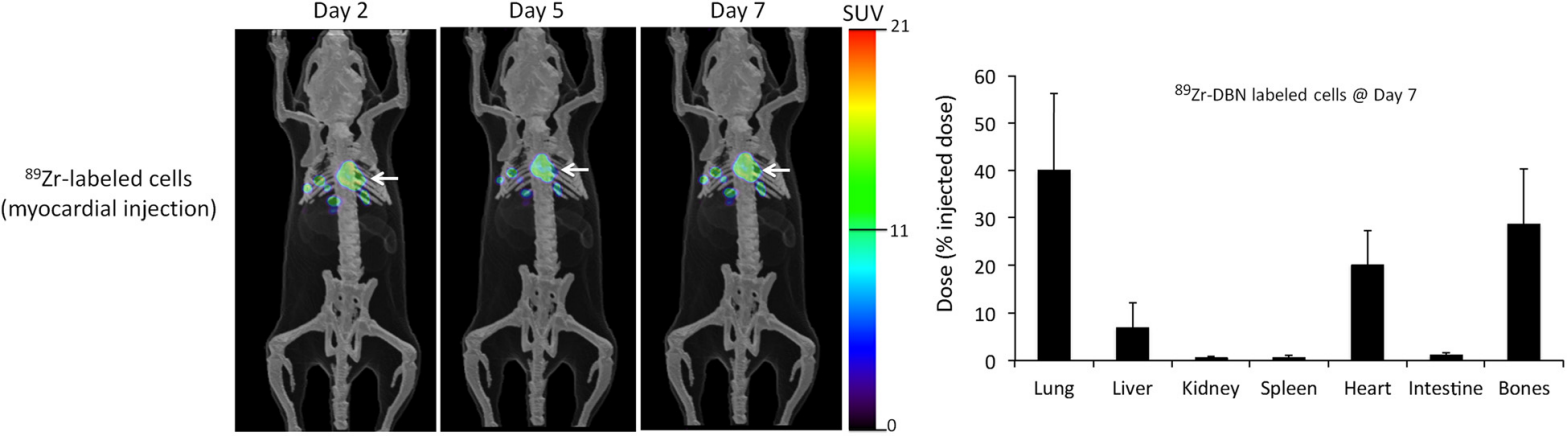
**Representative PET images and biodistribution data of** ^**89**^ **Zr-labeled hMSCs following myocardial delivery.** ^89^Zr-labeled hMSCs (2 × 10^5^ cells with radioactivity concentration approximately 0.37 MBq/10^6^ cells) were delivered to myocardium of an ischemia/reperfusion mouse model. Most of the radioactivity was distributed in the heart (arrow), lung, liver, and bones following myocardial delivery of ^89^Zr-labeled hMSCs. Values in graph are shown as mean ± standard deviation, *n* = 5.

Following myocardial delivery, ^89^Zr-labeled hMSCs (approximately 19.5% ± 9.5%) were retained for 7 days in the heart (Figure 6). The remaining cells were concentrated in the lung, followed by the bones and liver. The higher uptake in the lung relative to the liver is consistent with the biodistribution of ^89^Zr-labeled hMSCs released into the circulation (Figure 5).

## Discussion

Various strategies have been employed in the past to label cells with imaging isotopes for non-invasive *in vivo* cell tracking for cell-based therapies and infection imaging. Among them, ^18^ F-FDG (for PET) [12-16] and ^111^In-oxine (for SPECT) [2-6], are the most widely used. Although ^18^ F-FDG is useful for assessment of immediate delivery of cells and early fate of cells (approximately first few hours), it is not suited for *in vivo* cell tracking after 24 h post-injection due to its short half-life and poor retention in cells. Inability of ^18^ F-FDG to allow cell tracking after 24 h limits its utility in cell-based therapies. For cell-based therapies, early engraftment period of 2 to 5 weeks post cell delivery is the most critical time period [20]. Therefore, imaging-based methods should be robust over this time frame to allow evaluation of various interventions for improving cell engraftment. The ability to monitor cells *in vivo* beyond 24 h is also of high importance for evaluation of infection using radiolabeled leukocytes. Conventional infection imaging protocols perform imaging at 1, 4, and 24 h post-injection to differentiate between inflammatory, acute infection, and chronic infection loci; however, in some patients, 48 h was necessary for reliable detection of infected lesions [21].

The use of ^111^In (*T*_1/2_ = 2.8 days) as a radiolabel for cell labeling allows longer observation periods for cell tracking but with lower spatial resolution of SPECT imaging. Cell labeling with ^111^In typically requires a lipophilic carrier molecule (e.g., oxine) for transporting the radiometal into cells [2-6]. After entering the cells, the radiometal then dissociates and gets trapped in the cell by binding to non-specific intracellular metal-binding proteins. The two major disadvantages of this approach are chemotoxicity of the lipophilic carrier molecule [22] and efflux of radiolabel from cells [2-6].

Recently, two groups (Charoenphun et al. [9] and Davidson-Moncada et al. [10]) reported synthesis of ^89^Zr-oxinate or ^89^Zr-oxine as a cell labeling reagent for PET-based cell tracking. As expected, both groups faced the problem of chemotoxicity and significant efflux of radioactivity from the cells post-labeling commonly associated with oxine-based labeling. Charoenphun et al. [9] showed reduced viability of ^89^Zr-oxine-labeled 5 T33 myeloma cells (from 93% to 76.3% ± 3.2% in the first 24 h) and significant efflux of radioactivity post-labeling (29% effluxed in 24 h). Davidson-Moncada et al. [10] also reported similar results with ^89^Zr-oxine-labeled human and rhesus macaques’ natural killer cells. They observed a broad range of viability of 60% to 100% in the radiolabeled cells over the first 24 h, which declined to 20% to 30% after 6 days. A significant efflux of radioactivity was also observed in these viable ^89^Zr-oxine-labeled cells, approximately 20% to 25% effluxed in the first 24 h and 70% to 80% of radioactivity was effluxed after 7 days of culture. These drawbacks associated with the ^89^Zr-oxine labeling method compromises its utility for PET-based monitoring of *in vivo* cell trafficking.

To improve the stability of the ^89^Zr radiolabel on cells, we proposed ^89^Zr-DFO-NCS (^89^Zr-DBN) as a labeling entity capable of forming covalent bonds with primary amines of cell surface protein (Figure 1). Since all cells express cell surface protein with exposed lysine residues and other primary amines, this strategy also provides a general labeling method to label a broad array of cells. The new strategy exploits both the strength of chelation of ^89^Zr by DFO with three hydroxamate groups (qualitative Zr-binding constant = approximately 10^31^) [23-26] as well as the inherent biostability of the thiourea bond that conjugates NCS group in ^89^Zr-DBN to primary amines of protein [27,28]. Furthermore, the labeling agent, ^89^Zr-DBN, is also expected to be well-tolerated by cells as opposed to toxic lipophilic carrier molecules like oxine, relying on the fact that DFO-NCS has been routinely used to conjugate DFO to IgG and IgM antibodies with no loss of antibody protein function [23,26-29]. The generality of the labeling target, along with the multiplicity of primary amines available on the cell surface, also avoids the specific targeting of highly sensitive processes that might affect cellular function or viability. In contrast to the previously noted ^89^Zr-oxine results [9,10], no efflux of radiolabel was observed from cultured cells labeled with ^89^Zr-DBN after repeated washing and culture with medium with 10% fetal bovine serum (FBS) out to 7 days. These data strongly argue for a covalent bonding of the radiolabel to the cells. Furthermore, in a subcellular fractionation study, essentially all ^89^Zr radioactivity was incorporated into the membrane bound protein fraction of the cells confirming the anticipated targeting of membrane protein. The targeting of cell-surface membrane protein also has the potential benefit of distancing the labeling agent from potentially sensitive sites in the cell. We found no evidence of chemotoxicity or radiotoxicity effects of ^89^Zr-DBN labeling of three cell types in the present study.

In this study, 30% to 50% labeling efficiencies were achieved with ^89^Zr-DBN in several cell types. The cell labeling yield for mMCs, hMSCs, and mDCs were 0.50 ± 0.10, 0.47 ± 0.10, and 0.39 ± 0.20 MBq/10^6^ cells for mMCs, hMSCs, and mDCs, respectively. This is the maximum load of radioactivity per 10^6^ cells that we could achieve in this study. In the clinical setting, approximately 10^8^ cells are typically delivered to patients. Based on the labeling yield obtained in this study, the amount of ^89^Zr radioactivity administered to a patient would be in the range 30 to 50 MBq (0.8 to 1.4 mCi), which is in the range of ^89^Zr radioactivity that is currently being used in patients with ^89^Zr-labeled antibodies.

After encouraging *in vitro* validation tests, we performed *in vivo* validations by investigating the biodistribution of ^89^Zr-labeled hMSCs after intravenous injection in athymic nude mice for 7 days post-injection. Trapping of MSCs in the lungs following intravenous injection is well documented [30,31]. Therefore, we expected major accumulation of ^89^Zr-labeled human MSCs in mouse lungs following intravenous injection with slow clearance. With time, cells were expected to dislodge from this physical pulmonary entrapment and distribute to other organs. As expected, the majority of intravenously injected ^89^Zr-labeled hMSCs were trapped in the lung (50% ± 27%) and the remainder was found in the liver (27% ± 19%) and bones (16% ± 5%) after 7 days post-injection, which are expected homing sites for injected mesenchymal stem cells after dislodging from the lung [32,33]. This was in contrast to the biodistribution of ^89^Zr(HPO_4_)_2_, which distributed primarily in the bone (59% ± 13%) and liver (32% ± 15%) but did not accumulate in lungs. The distinct biodistributions of ^89^Zr-labeled hMSCs and ^89^Zr(HPO_4_)_2_, together with the stability of radiolabel and lack of cytotoxicity, strongly support the robustness of the ^89^Zr-DBN-based cell labeling approach.

To further test the application of ^89^Zr-labeled hMSCs, we performed a stem cell engraftment study using a myocardial acute ischemia/reperfusion mouse model. ^89^Zr-labeled hMSCs were delivered to the myocardium of athymic mice following an acute myocardial ischemia/reperfusion insult. After 7 days post-delivery, ^89^Zr-labeled hMSCs were found in the heart (20% ± 7%), lung (40% ± 16%), bone (29% ± 11%), and liver (7% ± 5%). The observed retention in the heart is in accordance with previously published work on hMSC engraftment estimated by invasive quantitative PCR method in a similar rodent model [34].

Low levels of *in vitro* demetalation of ^89^Zr-DFO complexes (2% to 3%/week) in serum at 37°C have been reported [23,29], and clinical studies using ^89^Zr-DFO-labeled antibodies out to 7 days have yet to show significant bone uptake of ^89^Zr indicative of demetalation [35-39]. Our initial findings of distribution of ^89^Zr-labeled hMSCs in mouse models confirm the biostability of the radiolabel bound to the DFO moiety supporting further exploration of the ^89^Zr-DBN labeling method for monitoring stem cell engraftment and cell trafficking. Extension of this approach with the use of an alternative zirconium chelator, such as 3,4,3-[LI-1,2-HOPO] [40] may further improve the biostability of the labeling agent.

## Conclusions

The ^89^Zr-DBN labeling agent is shown to be a robust, general, and biostable cell labeling strategy for PET-based non-invasive *in vivo* cell tracking. To explore the full potential of this approach, more work is needed to test this strategy in various model systems and disease processes that are germane to cell trafficking and stem cell therapies. We have ongoing efforts to define the imaging sensitivity, biostability, and toxicity parameters as the limits are pushed toward higher ^89^Zr radioactivity loading of cells and longer observation periods *in vivo.*

### Compliance with ethical standards

1. Disclosure of potential conflicts of interest: no authors have affiliations that present financial or non-financial competing interests for this work.
2. Research involving human participants and/or animals:
  All procedures performed in studies involving human participants were in accordance with the ethical standards of the institutional and/or national research committee and with the 1964 Helsinki declaration and its later amendments or comparable ethical standards. The patient-derived mesenchymal stem cells were obtained in compliance with institutional ethical review board guidance.
  All applicable international, national, and/or institutional guidelines for the care and use of animals were followed. All procedures performed in studies involving animals were under approval and in accordance with the Ethical Standards of Mayo Clinic Institutional Animal Care and Use Committee.
3. Informed consent: informed consent was obtained from all individual participants included in the study.

## Abbreviations

^89^Zr(HPO_4_)_2_: zirconium hydrogen phosphate
Alpha MEM: alpha modified Eagle’s medium
Anti-CD45: antibody against cluster of differentiation-45 antigen
DBN: desferrioxamine-NCS
DFO-Bz-NCS: desferrioxamine-benzyl-sodium thiocyanate
DMEM: Dulbecco’s modified Eagle’s medium
DTPA: diethylene triamine pentaacetic acid
FBS: fetal bovine serum
FDG: ^18^ F-2-fluoro-2-deoxy-D-glucose
GM-CSF: granulocyte-macrophage colony-stimulating factor
HBSS: Hank’s Balanced Salt Solution
HEPES-KOH: (4-(2-hydroxyethyl)-1-piperazineethanesulfonic acid)-potassium hydroxide
HMPAO: hexamethylpropyleneamine oxime
hMSCs: human mesenchymal stem cells
IgG: immunoglobulin G
IgM: immunoglobulin M
iTLC: instant thin layer chromatography
K_2_CO_3_: potassium carbonate
K_2_HPO_4_/KH_2_PO_4_: dipotassium hydrogen phosphate/potassium hydrogen phosphate
MBq: megabecquerel
mDCs: mouse-derived dendritic cells
MIPs: maximal images projection
mMCs: mouse-derived melanoma cells
PET: positron emission tomography
PTSM: pyruvaldehyde-bis(N4-methylthiosemicarbazone)
Rf: retention factor
SPECT: single-photon emission computerized tomography
T_1/2_: half-life of radioisotope
